# Current methodologies available to evaluate the virulence potential among *Listeria monocytogenes* clonal complexes

**DOI:** 10.3389/fmicb.2024.1425437

**Published:** 2024-10-10

**Authors:** Mariana Sousa, Rui Magalhães, Vânia Ferreira, Paula Teixeira

**Affiliations:** Universidade Católica Portuguesa, CBQF - Centro de Biotecnologia e Química Fina – Laboratório Associado, Escola Superior de Biotecnologia, Rua Diogo Botelho 1327, Porto, Portugal

**Keywords:** listeriosis, virulence, risk assessment, CC, infection

## Abstract

*Listeria monocytogenes* is a foodborne pathogen that causes listeriosis in humans, the severity of which depends on multiple factors, including intrinsic characteristics of the affected individuals and the pathogen itself. Additionally, emerging evidence suggests that epigenetic modifications may also modulate host susceptibility to infection. Therefore, different clinical outcomes can be expected, ranging from self-limiting gastroenteritis to severe central nervous system and maternal-neonatal infections, and bacteremia. Furthermore, *L. monocytogenes* is a genetically and phenotypically diverse species, resulting in a large variation in virulence potential between strains. Multilocus sequence typing (MLST) has been widely used to categorize the clonal structure of bacterial species and to define clonal complexes (CCs) of genetically related isolates. The combination of MLST and epidemiological data allows to distinguish hypervirulent CCs, which are notably more prevalent in clinical cases and typically associated with severe forms of the disease. Conversely, other CCs, termed hypovirulent, are predominantly isolated from food and food processing environments and are associated with the occurrence of listeriosis in immunosuppressed individuals. Reports of genetic traits associated with this diversity have been described. The Food and Agriculture Organization (FAO) is encouraging the search for virulence biomarkers to rapidly identify the main strains of concern to reduce food waste and economical losses. The aim of this review is to comprehensively collect, describe and discuss the methodologies used to discriminate the virulence potential of *L. monocytogenes* CCs. From the exploration of *in vitro* and *in vivo* models to the study of expression of virulence genes, each approach is critically explored to better understand its applicability and efficiency in distinguishing the virulence potential of the pathogen.

## 1 Introduction

Within the genus *Listeria*, twenty-eight species are recognized; however, only two are considered pathogenic: *Listeria ivanovii* and *Listeria monocytogenes* (Raufu et al., 2022; Vázquez-Boland et al., 2001). *Both L. ivanovii* and *L. monocytogenes* can cause listeriosis, but the majority of cases are attributed to *L. monocytogenes* and only a few to *L. ivanovii.* Although much rarer than those caused by *L. monocytogenes* and *L. ivanovii*, *L. innocua* infections have been reported in humans and ruminants (Favaro et al., 2014; Moura et al., 2019; Perrin et al., 2003; Rocha et al., 2013; Walker et al., 1994). Moura et al. (2019) demonstrated the virulence potential of atypical haemolytic *L. innocua* strains.

Human listeriosis, primarily caused by the consumption of contaminated food, is a severe illness that can manifest in one of two forms: non-invasive gastrointestinal infection in immunocompetent individuals or invasive listeriosis in risk groups, including pregnant women and newborns, the elderly and immunocompromised individuals (Vázquez-Boland et al., 2001; World Health Organization & Food and Agriculture Organization of the United Nations, 2004). In the invasive form, the pathogen surpasses the blood–brain and placental barriers, resulting in septicaemia, meningitis, spontaneous abortion and stillbirth (Lecuit, 2005). In 2022, the European Union reported 2,738 confirmed cases of listeriosis, which is 50 times fewer cases than the predominant gastrointestinal infection reported in humans, campylobacteriosis. Among the surveyed zoonotic pathogens, L. monocytogenes had the highest rates of hospitalization (96%) and case fatalities (18.1%) (EFSA and ECDC, 2023). These highlights the gravity of this major public health issue in developed nations. In addition to posing a significant public health risk, contamination of foods with this pathogen leads to disruptions in production, distribution, and recalls. As a result, it is receiving considerable attention from the food industry and authorities due to the significant economic losses and food waste involved (Li et al., 2022).

**Figure 1:**
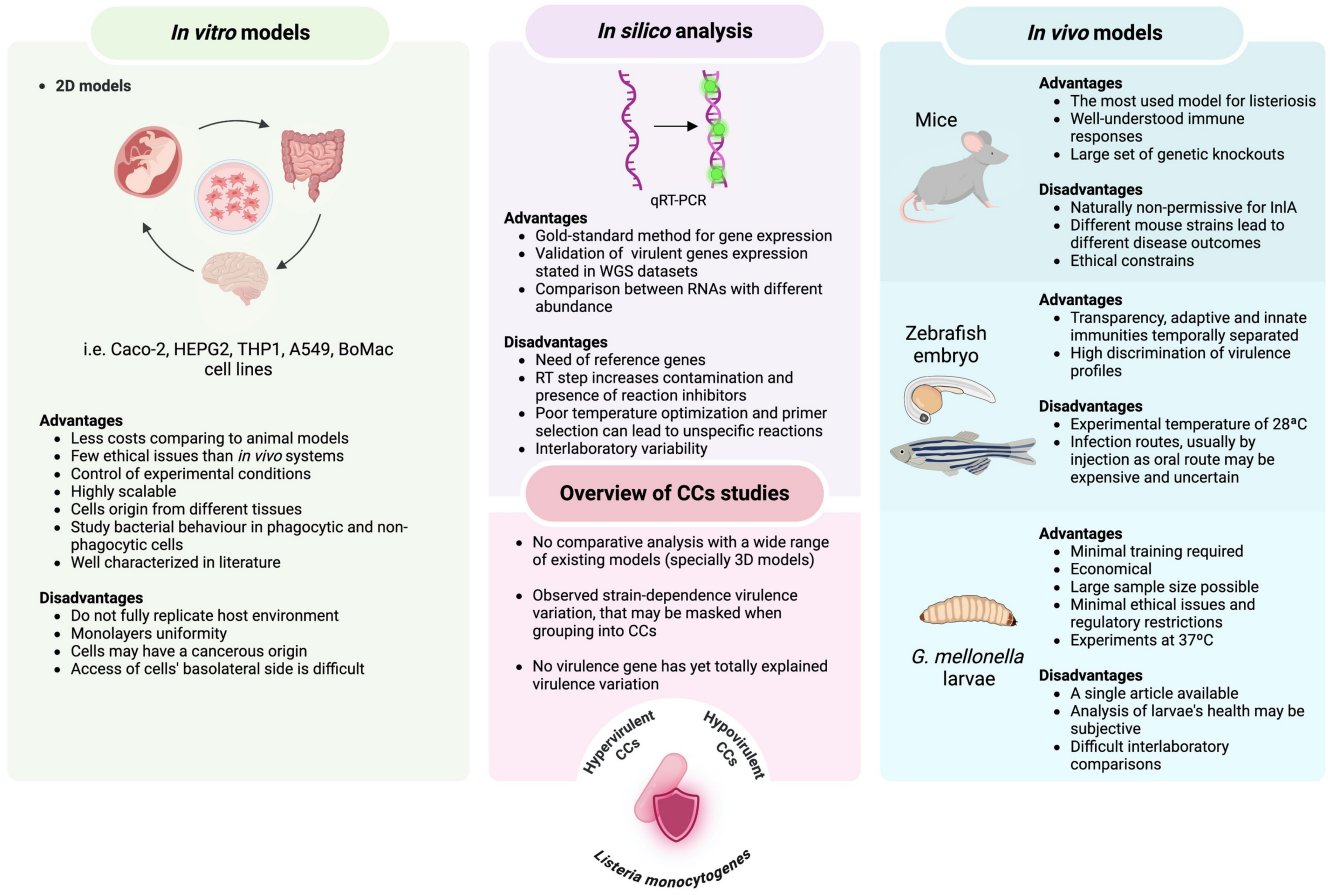
Overview of advantages and disadvantages of infection systems (both *in vitro* and *in vivo* models) and molecular approaches used to assess the virulence potential among *L. monocytogenes* clonal complexes.

The study of *L. monocytogenes* bacterial model is of undoubtable importance; however, scientific research cannot be directly performed in humans. The investigation of this foodborne pathogen infection in humans has been mainly through reported clinical cases, epidemiological data, genome analysis and the use of infection models. In addition, *L. monocytogenes* has relatively low incidence in humans and extended incubation periods can be challenging in listeriosis studies, hindering the identification of causing pathogen and contamination routes (Hoelzer et al., 2012; Vázquez-Boland et al., 2001). Although different methods have evolved to better characterize *L. monocytogenes*, this species is genetically heterogeneous and different typing methods (discussed below) can be used to subtype this species at different levels. Due to the great variety of typing methods available, comparative analysis between studies can be challenging (Koopmans et al., 2023). Additionally, the study of *L. monocytogenes* virulence potential can be conducted through different host species used as infection models, however, differences in the selected model, infection dose, incubation time, etc. can be difficult when comparing between studies. Several phenotypic and genotypic tools as well as *in vitro* and *in vivo* models have been used to evaluate the uneven virulence potential among distinct strains. Given the diversity of *L. monocytogenes* studies, it is challenging to define criteria that are universally objective, consistent and applicable. Therefore, in this review we aim to explore and analyse the current methodologies utilized for evaluating differences in the virulence potential among strains of distinct CCs (summarized in Figure 1), giving readers an overview of the available literature.

## 2 Typing of *Listeria monocytogenes*

Typing of *L. monocytogenes* has been essential in epidemiological studies of listeriosis, allowing for the establishment of clonal relatedness among collected isolates. Over the decades, the development and implementation of pheno-and geno-typing methods have made it possible to confirm outbreaks, trace sources of contamination and identify transmission routes within the food chain. Additionally, the increasing adoption of standardized typing methods has facilitated the establishment of effective national and international surveillance systems, enabling the monitoring of evolutionary trends and the generation of comparisons across different geographical regions. This has indubitably had a major influence on the responses and strategies of public health systems worldwide. On the other hand, these methods have massively enhanced our perception of the remarkable biodiversity within *L. monocytogenes* species and their distribution in different environments. The first method, largely employed in epidemiological studies, was based on the serological antigen structure of the bacterium, specifically on the agglutinating activity of somatic (O) and flagellar (H) antigens (Seeliger and Höhne, 1979; Seeliger and Langer, 1989). This method was gradually replaced by more expeditious methods – namely, a gel-based multiplex-polymerase chain reaction (PCR serogroup) that differentiates, between four major serogroups, including the serovars more frequently isolated from food and patients (> 98%, i.e., 1/2a, 1/2b, 1/2c, and 4b): serogroup IVb (comprising serovars 4b, 4d, 4e), serogroup IIa (comprising serovar 1/2a, 3a); IIb (comprising serovars 1/2b, 3b, 7); and serogroup IIc (comprising serovars 1/2c, 3c) (Doumith et al., 2004). Later, a real-time triplex-PCR assay that differentiates these groups was made available (Vitullo et al., 2013). Although many *L. monocytogenes* serotypes have been discovered, three major serovars (1/2a, 1/2b, and 4b) are responsible for a substantial fraction of listeriosis cases (about 90 to 95% of human infections) (Schiavano et al., 2022).

Several typing methods have been used for multiple purposes, with genotypic methods being particularly highlighted due to their higher discriminatory power (e.g., amplified fragment length polymorphism (AFLP), multilocus variable-number tandem repeat analysis (MLVA) or ribotyping) compared to phenotypic methods. In the specific case of epidemiological studies, pulsed-field gel electrophoresis (PFGE), based on the analysis of DNA restriction patterns, has been considered the “gold standard” technique for typing *L. monocytogenes* for many years (Graves and Swaminathan, 2001). However, PFGE has some drawbacks, such as the difficulty of standardizing the analysis of fingerprints, which poses a challenge for inter-laboratory and inter-country comparisons. In addition, while it is valuable for assessing genetic relatedness between isolates, pinpointing sources of contamination and identifying outbreaks, it is not sufficient for establishing comprehensive phylogenetic relationships between strains. Sequence-based typing methods such as multilocus sequence typing (MLST) or multi-virulence-locus sequence typing (MLVST) are more appropriate for this purpose (Maiden et al., 1998; Salcedo et al., 2003; Zhang et al., 2004). Currently, MLST is widely used as a reference method to categorize the clonal structure of bacterial species and to define clonal complexes (CCs) of genetically related isolates, i.e., those descended from the same ancestor. In *L. monocytogenes*, MLST is based on the sequencing of seven housekeeping genes (*acbZ, bglA, cat, dapE, dat, ldh*, and *lhkA*), that allow the determination of sequence types (STs) (Bergholz et al., 2018; Orsi et al., 2011; Ragon et al., 2008). Additionally, Ragon et al. (2008) grouped these STs within CCs, with strains sharing at least six out of seven MLST alleles being assigned to the same CC. Currently, the preferred method for epidemiological and phylogenetic studies has shifted to whole genome sequencing (WGS), which has become more accessible to a broader range of laboratories due to technological advances and reduced costs (Gerner-Smidt et al., 2019). Whole genome sequencing of *Listeria* provides high-resolution data that not only allows phylogenetic relationships between strains to be determined, but also provides in-depth knowledge of the genomic structure of a given strain, including information on specific virulence factors and other genes that contribute to pathogenesis, as well as potential antibiotic resistance prediction (Hurley et al., 2019; Moura et al., 2024).

This species presents a diverse genetic pool and its virulence potential is very heterogeneous, resulting in an uneven capacity of strains to cause disease (Pyz-Łukasik et al., 2022). Currently, this species is divided into four major evolutionary lineages (I-IV), comparable to subspecies (Liu, 2006; Orsi et al., 2008; Rasmussen et al., 1995; Roberts et al., 2006; Ward et al., 2008; Wiedmann et al., 1997); lineage I includes serotypes 1/2b, 3b, 4b, 4d, and 4e and 7, and is significantly overrepresented in human listeriosis cases (Gray et al., 2004; Orsi et al., 2011); lineage II includes serotypes 1/2a, 1/2c, 3a, and 3c, prevalent among isolates from environmental samples, food, and animal listeriosis cases (Nightingale et al., 2005b; Sauders et al., 2006), and contribute significantly to sporadic cases of human listeriosis (Jeffers et al., 2001); and, lineage III and IV include serotypes 4a, 4c and atypical serotype 4b isolates, which are rare and are mainly associated with listeriosis in animals (Liu, 2006). Clonal complexes are grouped within lineages, for example, CC1, CC2, CC4 and CC6 (serotype 4b, lineage I) and CC121 and CC9 (serotypes 1/2a and 1/2c, respectively, lineage II) (Maury et al., 2016). A methodology for cloning *L. monocytogenes* and assessing potential human infectivity has been patented (WO2017009198A1).

More than one hundred CCs have been reported globally. The predominance of particular CCs is highly heterogeneous among different sources and regions. In 2011, Chenal-Francisque et al. (2011) characterized the genotypic profile of three hundred isolates collected from 42 countries on five distinct continents, and these isolates were distributed within 111 STs, assembled into only 17 CCs. This reinforces the idea that there is an irregular geographical distribution, with a few prevailing CCs (Chenal-Francisque et al., 2011; Wagner et al., 2022). However, these isolates were collected between 1933 and 2007, and it has been established that the distribution of CCs tends to change over time with some CCs, such as CC9, CC121, CC5, and CC6, emerging more recently (Bergholz et al., 2018). Information on STs/CCs associated with listeriosis outbreaks in European countries during the last decade are presented in Table 1. When molecular characterization of outbreak strains was not available from publications, additional information was collected from the Institut Pasteur MLST database.^1^ CC6 and CC8 were the two main CCs, accounting for 17.4% of the total of 23 outbreaks identified. Many studies across European countries have also reported that some clonal complexes, such as CC1, CC2, CC3, CC4, CC5, CC6, CC8, CC9, CC37, CC121, and CC388 are globally prevalent with some geographical disparities (Domínguez et al., 2023; Félix et al., 2022; Maury et al., 2016; Painset et al., 2019). To better characterize this heterogeneity between strains from different CCs, two independent terms have been established: CCs with a high frequency in human clinical cases are considered hypervirulent; conversely, CCs associated with food, persistence in food manufacturing environments, and with a lower frequency in human listerioses cases are considered hypovirulent (Maury et al., 2016). Therefore, CC1, CC2, CC4, and CC6 (lineage I) are considered to be hypervirulent clones since they are clinically related and mainly infect individuals with low or no comorbidities. Contrarily, strains belonging to CC9 and CC121 (lineage II), recognized as hypovirulent clones, are regularly isolated from food and food processing environments. The latter are often associated with individuals with a compromised immune system (Maury et al., 2016). There is also an intermediate classification for those clones that may be in transition from their host-associated lifestyle due to loss of virulence and acquisition of stress resistance genes (FAO and WHO, 2022). In 2018, Fritsch and co-workers also established three different levels of virulence among CCs and STs for risk characterization: hypovirulence, medium virulence, and hypervirulence. The latter, includes, in addition to the previously mentioned hypervirulent CCs, CC224, ST54, CC101 + 90, ST87, ST451, ST504, CC220, ST388, and CC207 (Fritsch et al., 2018). Hypovirulent CCs include CC9 and CC121, as well as CC11, CC19, CC31, CC193, CC199, CC204, and ST124.

**Table 1:** Reported listeriosis outbreaks in Europe for the last decade.

Year	Country	Source^a^	Sequence type/clonal complex	No. of cases	Deceased^b^	^c^References
2009	Denmark	Beef meat – meals-on-wheels delivery	ST9/CC9	8	2	Smith et al. (2011), Jensen et al. (2016), and Moura et al. (2016)
2009–2010	Austria, Germany, Czech Republic	“Quargel” cheese	ST398/CC398 ST403/CC403 ST777/CC403	34	8	Fretz et al. (2010a,b), Harter et al. (2019), and Chen et al. (2016)
2009–2012	Portugal	Cheese	ST388/CC388	30	2	Magalhaes et al. (2015) and Ferreira et al. (2018)
2011	Belgium	Hard Cheese	ST37	12	4	Yde et al. (2012)
2011–2013	Austria and Germany	Unaged soft cheese and shrink-wrapped deli meat – plausible suspects	ST398/CC398 ST403/CC403	7	2	Schmid et al. (2014) and Moura et al. (2016)
2012–2015	Germany	Smoked pork belly	ST8/CC8	66	6	Ruppitsch et al. (2015) and Kleta et al. (2017)
2013–2014	Denmark	Deli meat products (mainly spiced meat roll)	ST224	41	17	Kvistholm et al. (2016)
2013–2014	Switzerland	RTE salad	ST4/CC4	31	4	Tasara et al. (2015) and Stephan et al. (2015)
2013–2015	Denmark	cold smoked salmon	ST391	10	4	Gillesberg et al. (2016)
2013–2015	Denmark	cold smoked halibut and trout	ST6	10	3	Gillesberg et al. (2016)
2013–2018	Germany	RTE meatballs	CC5 CC7	83	5	Wilking et al. (2021) and Lüth et al. (2020)
2014–2019	Multicountry outbreak: Estonia, Denmark, Finland, France, Sweden	Fish products	ST1247/CC8	22	5	Mäesaar and Roasto (2020), ECDC and EFSA (2019), and Mäesaar et al. (2021)
2014–2019	Germany	RTE meat sausages	ST8	39	18	Lachmann et al. (2021) and Fischer et al. (2021)
2015	Italy	Cheese	ST29/CC29, ST1/CC1, ST7/CC7, ST398/CC398	6	1	Comandatore et al. (2017)
2015–2017	Cross-border: Denmark and France	Cold-smoked salmon	ST8/CC8	7	1	Schjørring et al. (2017)
2015–2017	Austria	Meat processing company (sliced pizza ham)	ST155/CC155	7	n/d	Pietzka et al. (2019)
2015–2018	Multicountry outbreak: Austria, Denmark, Finland, United Kingdom, Sweden	Frozen vegetables (corn)	ST6/CC6	47	9	EFSA and ECDC (2018), EFSA et al. (2020), and McLauchlin et al. (2021)
2016	Italy	Sliced cold beef ham	ST1/CC1	40	n/d	Maurella et al. (2018)
2018	Austria	Liver pâté	ST4/CC4	13	n/d	Cabal et al. (2019)
2018–2019	Germany	Blood sausage	ST6/CC6	112	7	Halbedel et al. (2020) and Wilking et al. (2021)
2018–2020	Switzerland	Cheese	ST6/CC6	34	10	Nüesch-Inderbinen et al. (2021)
2019	Spain	Stuffed pork	ST388/CC388	207	3	Fernández-Martínez et al. (2022) and Domínguez et al. (2023)
2020–2021	Multicountry outbreak: Germany, Austria, Denmark, and Switzerland	Smoked rainbow trout filets	ST394	55	3	Halbedel et al. (2023)

a RTE, ready-to-eat.

b n/d, no data available.

c For a comprehensive overview of listeriosis outbreaks worldwide between 1969 and 2022, we refer to the recent review by Koopmans et al. (2023).

It is important to note that, although hypovirulent CCs such as CC9 and CC121 are mainly associated with food and food processing environments, cases of invasive listeriosis caused by these CCs have also been reported. For instance, CC121 was considered the second most common CC isolated from human clinical cases in Norway and in France (Fagerlund et al., 2022; Maury et al., 2016).

Despite the potential ability to predict the risk of a specific strain of *L. monocytogenes* causing disease after consumption of contaminated food, most regulatory authorities worldwide take action when any *L. monocytogenes* is found in ready-to-eat (RTE) food that is capable of supporting growth, regardless of its strain characteristics. This approach is recommended by the Food and Agriculture Organization (FAO) and the World Health Organization (WHO) (2022), although in some countries risk managers are permitted to use information on *L. monocytogenes* subtypes to guide risk management decisions. However, the FAO and the WHO encourage the search for other virulence markers to predict, based on genetic virulence profiles (CCs characterization) (FAO and WHO, 2022). The discovery of one or multiple biomarkers that would allow to predict the real virulence potential of a given strain, and a clear distinction between hypo-and hypervirulence would be of great value to reassess the risks associated with different *L. monocytogenes* strains and to develop appropriate policies that neither overstate nor underestimate the risk posed by each strain. Ultimately, this finding would also contribute massively to the reduction of costs associated with the recall and destruction of contaminated food products and to reduced food waste and its social and economic consequences.

## 3 Putative virulence biomarkers (core and accessory genome)

The *L. monocytogenes* infection cycle comprises various steps: adhesion and invasion, lysis and escape from the vacuole, cytosolic multiplication, actin-tails polymerization, spread to neighbouring cells, and rupture of a double-membrane vacuole (Luque-Sastre et al., 2018; Pizarro-Cerdá et al., 2012). Some virulence genes are important for infection, such as, InlA-E-cadherin and/or InlB-C-Met (*L. monocytogenes* internalins-host receptors) for invasion, listeriolysin O (LLO) and phospholipases A and B (PlcA and PlcB) for both primary and double-layer vacuoles disruption, ActA for actin tail polymerization and intracellular motility (Quereda et al., 2021; Radoshevich and Cossart, 2018).

Considering all the above information, a detailed investigation regarding the putative virulence markers linked to both hyper-and hypovirulence is still ongoing, and some interesting findings have been reported. Regarding the core genome, inlA is normally present and expressed as a full-length form within clinical isolates (Lecuit et al., 2001). Premature stop codons mutations (PMSCs) have been found in the inlA gene, resulting in a truncated non-functional internalin in food isolates. In some studies, these PMSCs have been found amid strains from hypovirulent CCs, such as CC9 and CC121, and thus it is hypothesized that in some way, the lower virulence potential of these strains can be justified by the InlA truncation, leading to a reduced capacity to cross the intestinal barrier (Jacquet et al., 2004; Lachtara et al., 2022; Moura et al., 2016). The significant role of InlA-mediated crossing of *L. monocytogenes* through the intestinal barrier has been described. However, some studies have showed that the inoculation of Δ*inlA* mutants still resulted in *L. monocytogenes* infection (Bierne et al., 2002; Bou Ghanem et al., 2012; Disson et al., 2008). Therefore, it was hypothesized that *L. monocytogenes* employes alternative routes to cross the intestinal barrier. Besides the M cell-mediated translocation in Peyer’s patches, Drolia et al. (2018, 2024) showed that the linkage between Listeria adhesion protein (LAP) and its surface receptor Hsp60 promotes cell disruption by using the cell innate system, consequently leading to bacterial translocation (Drolia et al., 2024; Drolia et al., 2018). These studies have shown that *L. monocytogenes* can cross the intestine through InlA-independent routes, which could explain the isolation of strains belonging to hypovirulent CCs (normally associated with the production of truncated inlA) in clinical cases. However, to our knowledge no comparative studies have investigated LAP or other InlA-independent invasion factors as putative candidate to distinguish hyper-or hypovirulent strains of *L. monocytogenes*.

All strains of *L. monocytogenes* carry the Listeria Pathogenicity Island 1 (LIPI-1), which clusters several fundamental genes for *L. monocytogenes* pathogenicity (Moura et al., 2016; Vázquez-Boland et al., 2001). These include the hly gene, which encodes a hemolysin – LLO – that provides the capacity to lyse erythrocytes. As mentioned above, this toxin can form a pore and allow the bacteria to escape from the internalization vacuole; thus, this virulence factor is detrimental to the virulence of *L. monocytogenes*. Another important virulence factor is PrfA, known as the main regulator of virulence genes in *L. monocytogenes*, such as the *prfA*, *actA* and *hly* genes. However, some studies have reported the existence of non-hemolytic *L. monocytogenes* strains, belonging to both lineages I and II, that have mutations in either the *prfA* or *hly* genes, and consequently a lower virulence potential (Maury et al., 2017).

Regarding the accessory genome, the pathogenicity island LIPI-3 carries eight genes. Listeriolysin S (LLS) encoded by *llsA*, functions as a bacteriocin with the capacity to modify the composition of the intestinal microbiota by eliminating or hindering the growth of neighbouring bacteria. This virulence cluster is often present within lineage I isolates, especially those from CC1, CC2 and CC6 – constituting a potential marker of hypervirulence (Cotter et al., 2008; Moura et al., 2016; Quereda et al., 2016). Additionally, Maury et al. (2016) identified a novel virulence cluster termed LIPI-4, which aggregates six genes that encode a cellobiose family phosphotransferase system (PTS). This gene cluster is strongly associated with strains of CC4, which are highly relevant to human brain and placental infections (Maury et al., 2016; Moura et al., 2016). Furthermore, it was thought that this pathogenic island was exclusively related to CC4 strains, but isolates from CC87 in China also displayed this locus (Wang et al., 2019; Zhang et al., 2020). These findings suggest that this could be a putative marker of hypervirulence, although it was found that this island was also present in *L. innocua* – a non-pathogenic species – and thus its role in hypervirulence is still controversial, reinforcing the need for further studies. Another intriguing gene is *lmo2776*, which acts as a bacteriocin and plays an important role in modulating the intestinal microbiome, mainly targeting *Prevotella copri* – a common gut commensal that has the capacity to modify the intestinal mucus layer and potentially intensify gut infection. The critical aspect is its significant presence in lineage I strains compared to its low frequency in lineage II strains. Curiously, deletion of *lmo2776* resulted in a better spread of the bacteria to the liver and spleen – the primary target organs of *L. monocytogenes* after crossing the intestinal barrier. This can be explained by the capacity of *L. monocytogenes* to discriminate between *P. copri*, preventing exorbitant inflammation and leading to longer periods of infection (Rolhion et al., 2019).

## 4 Models to study *Listeria monocytogenes* clonal complexes

### 4.1 *In vivo* infection models

In virulence studies, both pathogen characteristics and host physiology and anatomy must be considered, as microbial infections result from interactions between pathogens, hosts and the surrounding environment (Prescott, 2022). Preclinical trials using *in vivo* and *in vitro* biological models, have provided valuable insights into host-pathogen interactions (Anju et al., 2020; Khan et al., 2018). *In vivo* systems, used for various purposes from drug development to investigating physiological processes, complement *in vitro* studies by providing a more comprehensive understanding of biological responses. However, neither system alone is sufficient to make absolute predictions (Khan et al., 2018). Some of these models will be detailed in the following sections (Table 2).

**Table 2:** Infection models used to study virulence potential among *L. monocytogenes* CCs.

Model		CC/ST/serotypes (no. of strains)	Source	Year	Reference/control strains	Reference
*In vivo*	Mice	CC1 (*n* = 3)	Ganges river, agricultural soil and human placenta bit	2017	ATCC19115 and MTC-C1143	Soni et al. (2017)
		CC388 (*n* = 1), CC1 (*n* = 1), CC4 (*n* = 4)	Meat and retail products – ooutbreak strain in Spain (CC388)	2019	ATCC19115 (CC2)	Domínguez et al. (2023)
		CC1 (*n* = 1), CC4 (*n* = 1), CC6 (*n* = 1)	N/A	2022	EGDe (CC9)	Maudet et al. (2022)
	*G. mellonella* larvae	CC1 (*n* = 6), CC6 (*n* = 5), CC7 (*n* = 9), CC9 (*n* = 4), CC14 (*n* = 6), CC37 (*n* = 1), CC204 (*n* = 3)	Bovine (*n* = 16), human (*n* = 12), goat (*n* = 2), faeces (*n* = 1), rabbit (*n* = 1), silage (*n* = 1)	2019	–	Cardenas-Alvarez et al. (2019)
	Zebrafish	CC121 (*n* = 1), CC9 (*n* = 2), CC31 (*n* = 1), CC3 (*n* = 1), ST213 (*n* = 1), CC218 (*n* = 1)	Food (meat, vegetables), environmental swab,	2019	EGDe (CC9)	Hurley et al. (2019)
		CC1 (*n* = 9), CC2 (*n* = 6), CC4 (*n* = 6), CC6 (*n* = 4), CC8 (*n* = 5), CC9 (*n* = 10),	Human (*n* = 14), food (meat, milk cheese, RTE-salads, plant associated, ham) (*n* = 25), rabbits (*n* = 1)	2022	–	Muchaamba et al. (2022)
*In vitro*	Caco-2 cells	CC1 (*n* = 3), CC7 (*n* = 1), CC9 (*n* = 1), CC31 (*n* = 1), CC101 (*n* = 1), CC121 (*n* = 2)	Human (blood, CSF) (*n* = 3) and food (head cheese, fresh salami, salami, spit roasted pork) (*n* = 6)	2022	*L. innocua* ATCC 33090	Schiavano et al. (2022)
		CC1 (*n* = 3), CC2 (*n* = 2), CC3 (*n* = 3), CC315 (*n* = 3), CC5 (*n* = 3), CC121 (*n* = 5), CC14 (*n* = 3), CC19 (*n* = 4), CC403 (*n* = 3), CC415 (*n* = 3), CC7 (*n* = 6), CC8 (*n* = 4), CC9 (*n* = 4)	Food (salmon and meat)	2022	EGDe	Wagner et al. (2022)
		CC7 (*n* = 5)	Salmon and meat processing environment, dairy	2024	EGDe	Møretrø et al. (2024)
		CC1 (*n* = 1) and respective mutant strains	Rhombencephalitis in cattle	2017	EGDe, *L. innocua* (CCUG15531)	Rupp et al. (2017)
	HEPG2 hepatocytes	CC14 (*n* = 3), CC9 (*n* = 2)^a^, CC121 (*n* = 1)^a^	Salmon	2022	EGDe	Wagner et al. (2022)
		CC7 (*n* = 5)	Salmon and meat processing environment, dairy	2024	EGDe	Møretrø et al. (2024)
	Macrophage-like THP1 cells	CC14 (*n* = 3)	Salmon	2022	EGDe	Wagner et al. (2022)
		CC7 (*n* = 5)	Salmon and meat processing environment, dairy	2024	EGDe	Møretrø et al. (2024)
	A549 cells	CC388 (*n* = 1), CC1 (*n* = 1), CC4 (*n* = 4)	Meat and retail products – outbreak strain in Spain (CC388)	2019	ATCC19115 (CC2)	Domínguez et al. (2023)
	Macrophage-like BoMac cells	ST1, ST4, ST412, ST18, ST37	Cattle	2016	–	Dreyer et al. (2016)
		CC1 (*n* = 1) and respective mutant strains	Rhombencephalitis in cattle	2017	EGDe, *L. innocua* (CCUG15531)	Rupp et al. (2017)
	Human macrophages differentiated from peripheral blood monocytes.	EGDeΔinlB supplemented with idInlB_CC1_^b^, idInlB_CC7_ and idInlB_CC9_	–	2023	–	Chalenko et al. (2023)
	Intestinal organoid from mice	1/2a (*n* = 1), 4a (*n* = 1)	–	2022	–	Zhou et al. (2022b)
Molecular approaches	RT-qPCR	CC1 (*n* = 1)	Rhombencephalitis in cattle	2017	EGDe, *L. innocua* (CCUG15531)	Rupp et al. (2017)
		CC14 (*n* = 2), CC9 (*n* = 2)^a^, CC121 (*n* = 1)^a^	Salmon	2022	EGDe	Wagner et al. (2022)

N/A, not available; CSF, cerebrospinal fluid.

a Reconstructed strains with inlA gene.

b Receptor-binding domains of InlB.

In order to improve human health research, both mammalian and non-mammalian models are used due to ethical constraints with experiments involving humans (World Medical Association, 2013). The broad host range of *L. monocytogenes* allows the use of various animal models, such as *Drosophila melanogaster* (fly), *Galleria mellonella* (moth), *Caenorhabditis elegans* (nematode), *Mus musculus* (mouse), *Cavia porcellus* (guinea pig), *Oryctolagus cuniculus* (rabbit), among others (Anju et al., 2020; Prescott, 2022) – some of which will be discussed further. Animal models offer advantages that make them invaluable for human health research: they have identical biological processes, anatomical similarities (especially in vertebrates animals) – which are difficult to replicate in *in vitro* systems – compatible diseases such as cancer and diabetes, short life cycle and some can be easily genetically transformed to acquire some fundamental characteristics to express the disease phenotype (Kiani et al., 2022). Additionally, *in vivo* models are essential because they possess some unique characteristics when compared to *in vitro* models, for instance, the immunity associated with commensals and the intestinal mucosa throughout infection (Eng and Pearson, 2021). Depending on the final objective of the study, several aspects must be considered when selecting the ideal animal model: (1) the pathogen should have a similar tissue and cell affinity as in humans; (2) it should reveal the identical observable disease outcome and immunopathological harm; and (3) it should be susceptible to genetic manipulation (Lecuit, 2007). In addition to animal features, to study *L. monocytogenes* virulence, understanding listeriosis pathophysiology is crucial to select the adequate animal model. As already mentioned, *L. monocytogenes* can cross the intestinal, blood–brain and placental barriers. Therefore, pregnant, non-pregnant and geriatric animal models have been used in the study of *L. monocytogenes* pathogenesis, this was exhaustively described by Hoelzer et al. (2012). Animal models have played crucial roles in the characterization the virulence of *L. monocytogenes.* Generally, insightful data about the different pathways of bacterial translocation through host’s defensive barriers, the exploitation of host’s immunity to improve disease, performance of dose-dependent assays, the complex host immune responses to infection, the species specificity, virulence factors and strains virulence potential have been emerged from animal models studies (Hoelzer et al., 2012; Koopmans et al., 2023; Lecuit, 2007; Maury et al., 2016; Tsai et al., 2013). To our knowledge, an ideal animal model for listeriosis has not been established. Continuous new insights into animal physiology have increase the possibilities for infection systems, yet no single animal model completely aggregates the desirable characteristics to study human listeriosis. Therefore, the selection of an *in vivo* system is according to the specific objectives of the research being conducted.

Although animal models bring unquestionable insights into the study of infectious diseases, their extensive and indiscriminate use is strongly condemned by the European commission. This authority bases its policy on the Three R’s principle (Replacement, Reduction and Refinement), which aims to replace the use of animals with non-animal strategies, to use a reduced number of animals per experiment without compromising the ultimate aim of the research, and to improve practices that contribute to the welfare of animals from birth to death. When animal replacement is not possible, the use of animals must follow strict guidelines set out in EU Directive 2010/63/EU (European Parliament, 2010; Zuang et al., 2024).

#### 4.1.1 Mammalian models (mice)

The establishment of Robert Koch postulates to determine the etiological agent of an infectious disease, marked the inception of using mammalian species, phylogenetically related to humans, as healthy susceptible models (Kaito et al., 2020; Short and MacInnes, 2022).

*Listeria monocytogenes* is a ubiquitous microorganism, which enables it to infect a wide range of animals (Kammoun et al., 2022). However, in addition to humans, it mainly causes disease in ruminants, which, in an immediate and logical thought, should be the primary models to study listeriosis. However, this brings up many limitations. Thus, mice are the standard *in vivo* model to study listeriosis due to their size, ease of breeding and reproduction, rapid acclimation to confinement and an equivalent physiology when compared to humans (Lecuit, 2007). Commonly, mice are intravenously infected with the pathogen, and the role of some virulence factors, such as ActA and LLO, have emanated from this technique (Disson et al., 2009). Although mice are widely used in *L. monocytogenes* studies, the efficacy of oral infection is low due to the species-specific associated of with mammalian cells. As mentioned above, InlA binds to the E-cad receptor, which is a specific linkage for each species, depending on its 16^th^ amino-acid type. Permissive species, such as guinea pigs, rabbits, humans and gerbils, have a proline in this position while non-permissive species have a glutamic acid – mice and rats have the glutamic acid and, consequently do not allow InlA binding (Lecuit et al., 1999). On the other hand, InlB naturally binds to C-Met in mice, humans and gerbils (Khelef et al., 2006). Theoretically, animals that naturally possess the imperative requirements to be bound to *L. monocytogenes* internalins, such as ruminants, non-human primates, and gerbils, should be selected to study listeriosis (Kammoun et al., 2022). Nonetheless, the ethical hurdles do not allow their wide application, so humanized mice have surged to overcome this limitation (Disson et al., 2008; Lecuit et al., 2001). Additionally, a “murinized” *L. monocytogenes* strain was developed to interact more closely with mouse E-cadherin. This modification involved altering the *inlA* gene in the *L. monocytogenes* EGDe strain to successfully infect wild-type mice (Wollert et al., 2007). Although this species-specific limitation was overcome, it was further discovered that the altered InlA was able to interact with both E-cadherin and N-cadherin in mice, luminally accessible in goblet and M-cells respectively, leading the bacteria to target both cells, increasing gut inflammation and consequently, hindering the capacity of *L. monocytogenes* to spread in the host (Tsai et al., 2013).

Currently there has not been a published comparative analysis of clonal complexes and their virulence in some animal models, such as gerbils, non-human primates, guinea pigs or rats. Although gerbils are permissive to both receptors, their use in listeriosis studies is limited. This may be related to the decreased sensitive to oral infection with *L. monocytogenes* when compared to other models, insufficient characterization when compared to mice and guinea pigs, absence of genetic models, and limited specific reagents and antibodies. The guinea pig infection model is advantageous in maternal-fetal studies as its placenta is the most comparable to human placenta among all rodents and has equivalent placental tropism. However, its narrow use may be related not only to species-specificity but also due to different disease symptoms from human listeriosis, with weak central nervous system tropism. Guinea pigs also present long gestation periods compared to mice, lack of gene deletion and transgenic models, and their larger size is more costly, limiting the number of animals per experiment. Despite the similarities of rats to mice, this infection model has shown low susceptibility to infection, requiring high infection doses to provoke disease. The use of non-human primates, as expected, is limited due to extended gestation periods, reduced number of available animals per study, and limited gene libraries compared to mice. Additionally, all these models are most costly when compared to mice (Cossart, 2011; D'Orazio, 2014; Eallonardo and Freitag, 2024; Hoelzer et al., 2012; Khelef et al., 2006; Roulo et al., 2014; Yan et al., 2023).

Considering this, very few articles have employed mice to investigate this phylogenetic association, either directly or indirectly (Domínguez et al., 2023; Maudet et al., 2022; Soni et al., 2017). Although the objective was not to compare strains from different CCs, Soni et al. (2017) inoculated three strains from CC1 in mice and observed a varying disease-causing capacity. One strain did not kill any mouse, while the other two presented 60 and 100% relative virulence. This highlights that although CCs are a more thorough classification, strains within a single CC can exhibit different virulence potentials. They also observed that the three strains harboured the major virulence genes, with the strain showing the lower pathogenicity presenting mutations in crucial virulence factors, such as listeriolysin O. However, no conclusion has been reached as to which mutation or genes better explain this unequal pathogenicity between phylogenetically close strains (Soni et al., 2017). Furthermore, in 2019, a large outbreak of listeriosis occurred in the Andalusian region, causing 207 cases, which was later associated with the strain ST388 from CC388 (Ministerio de Sanidad Consumo y Bienestar Social de España, 2019). Domínguez and her colleagues proceeded to investigate the virulence potential of this strain by comparing it with other strains from hypervirulent CCs (CC1 and CC4). *In vivo* infection assays were performed, and mice were infected intravenously with four strains (reference ATCC^®^ 19115™, CC1, CC4, and CC388 strains). The results showed no significant differences between the CC388 strain and the other hypervirulent strains, as CC4 and CC388 isolates exhibited identical infection and spread ability (Domínguez et al., 2023). Maudet et al. (2022) selected strains from CC1, CC4, and CC6 – previously characterized as highly neuroinvasive CCs (Maury et al., 2016) – to perform infection assays in humanized KIE16P mice. The comparative analyses performed between these hypervirulent CCs and EGDe strain (CC9), corroborated the increased capacity of hypervirulent CCs to invade mice brains. Additionally, gene expression assays showed that hypervirulent strains presented upregulated levels of the *inlAB* operon, when compared to EGDe. Throughout these experiments different ∆*inlB* mutant strains were constructed to validate its relevance in the neuroinvasion capacity of *L. monocytogenes*. Despite the reduced neuroinvasion levels of EGDe when compared to CC4 strains, this study showed that whether using hypovirulent or hypervirulent strains, the *inlB* gene deletion reduced bacterial loads in the brain, confirming the need of overexpressing the *inlB* gene in *L. monocytogenes* neuroinvasiveness. Furthermore, the authors reported that InlB has immunosuppressive properties that are crucial to protect infected cells from host immune responses, resulting in an increase of infected monocytes’ lifespan and *L. monocytogenes* propagation to the brain. Additionally, as hypervirulent strains exhibit overexpression of *inlB* and are mainly associated with infections in immunocompetent individuals, this article highlights the need to continuously study hypervirulent CCs to improve our perspective regarding the bacterial factors employed in *L. monocytogenes* infection mechanism. Altogether, these finding showed that regardless of some reports suggesting strain-dependence in *L. monocytogenes* virulence studies, strains from hypervirulent CCs confer a significant concern to human health with distinct virulence factors that allows them to evade the host immune system. Moreover, mice models have proven to be a reliable tool to study *L. monocytogenes* infection cycle in mammals and we believe they will continue to be useful in future works.

#### 4.1.2 Non-mammalian model organisms

Although mammalian models are the paradigm for studying host-pathogen interactions, they still present many obstacles, such as ethical issues due to animal welfare, high costs, adequate facilities and differentiated training requirements. Therefore, alternative models are needed for *in vivo* experiments that are less costly, easier to manipulate, with a short life cycle and are ethically acceptable. The complexity and relevance of these models lie between the sophisticated humanized mice and the simplicity of *in vitro* approaches (Lecuit, 2007). A variety of invertebrate and vertebrate models have been used to study the virulence potential of pathogens and the host immune response (Ahlawat and Sharma, 2022; Mylonakis et al., 2007). In *L. monocytogenes* studies, we highlight the use of *G. mellonella* larvae, *Drosophila melanogaster*, *Caenorhabditis elegans* and *Danio rerio* (zebrafish), which have given valuable insights in the study of listeriosis. For instance, *C. elegans* model was previously used to evaluate the effects and toxicity of antimicrobial or antibiofilm substances in host-pathogen interactions and study nitrogen metabolism of *L. monocytogenes* after nematodes gut colonization (Kern et al., 2016; Muthulakshmi et al., 2022; Silva et al., 2015; Sivaranjani et al., 2016). The pathogenesis of *L. monocytogenes* has also been explored in *D. melanogaster* model, focusing in host immune system modulation, fly’s metabolism alterations upon infection, association between the bacterial growth dynamics and host’s genotypes (Chambers et al., 2012a,b; Hotson and Schneider, 2015; Mansfield et al., 2003; Taillebourg et al., 2014). Besides the widely use of both *C. elegans* and *D. melanogaster* models in the study of pathogenic bacteria, to our knowledge there are no comparative studies between *L. monocytogenes* CCs. In the fly model this can be related to the fact that the favourable temperature to flies is between 22 and 25°C, however, listeriosis studies are mainly conducted at 30–37°C and its inadequacy to distinguish between avirulent and virulent *Listeria* spp. after bacterial injection into the flies thorax (Jensen et al., 2007). On the other hand, *C. elegans* limited use may be associated to the fact that the deletion of some bacterial virulence genes (i.e., ActA) did not affect nematode’s death and that the *C. elegans* intestine architecture may be different from mammals, since neither cell junction during cell extrusion or in goblet cells lumen are common in this nematode (Balla and Troemel, 2013; Thomsen et al., 2006). Considering this, we focused on both zebrafish and waxworms to give an overview about the study of virulence potential between *L. monocytogenes* strains/CCs in non-mammals.

##### 4.1.2.1 Insect models

In the past, it was thought that insects were not a good *in vivo* model to study microorganisms that cause disease in humans since they are not phylogenetically close. However, they share a few physiological aspects with humans. Human pathogens present an analogous virulence capacity in humans and insects, with similar virulence factors involved (Mansfield et al., 2003; Martinez et al., 2017; Mukherjee et al., 2010; Tsai et al., 2016). In addition, the pathogen follows similar infection cycle steps in both hosts. Consequently, insects have evolved some defence mechanisms that are shared between mammals and insect hosts, for instance, the innate immune system with physical and phagocytic barriers, that have a homologous function (Kemp and Massey, 2007; Peterson et al., 2008). However, insects lack the capacity to develop an adaptive immune response, which is a common feature in vertebrates (Ahlawat and Sharma, 2022; Tsai et al., 2016). Hence, insects as host models have been a convenient alternative to mammals for infectious disease research.

###### 4.1.2.1.1 *Galleria mellonella* as an infection model

Experiments with *Galleria mellonella* larvae have been carried out for some time, with increasing interest in recent year as a potential surrogate model to explore pathogen infections (Dinh et al., 2021). Besides being small, cheap, short life cycle, easy to maintain and to obtain in large numbers, it is also adapted to temperatures from 25°C to 37°C – the optimum growth temperature for the vast majority of human pathogens (Dinh et al., 2021; Mylonakis et al., 2005). The wax worm is selected stage to be utilized as a model, with infection normally occurring by injection, which requires minimal training (Singkum et al., 2019; Tsai et al., 2016). Its whole genome has recently been sequenced, enabling the search for further novel insights (Lange et al., 2018). Moreover, these insects possess a relatively advanced innate immune system, comprising two main components – the cellular and humoral immune response. The primer is composed of hemocytes – phagocytic cells that prevail in the hemolymph, and they are also capable of encapsulation and nodulation of pathogens. The humoral response results from the production of lytic enzymes, antimicrobial peptides (AMPs), opsonins and melanin upon microbial exposure (Boman and Hultmark, 1987; Kavanagh and Reeves, 2004; Pereira et al., 2018). It has been reported that *G. mellonella* larvae infected with *L. monocytogenes* are prone to produce AMPs such as galiomycin, lysozyme, gallerimycin, insect metalloproteinase inhibitor (IMPI) and cecropin D (Mukherjee et al., 2011; Mukherjee et al., 2010). Beside the analysis of host’s immune modulation through hemocytes enumeration and variations in AMPs expression, the melanization, survival capacity, development of cocoon, motion ability can be evaluated in infected larvae with *L. monocytogenes* (Kavanagh and Sheehan, 2018).

*Galleria mellonella* has been utilized as an infection model to study the virulence potential of *L. monocytogenes* through comparative studies with different *Listeria* species or comparisons between *L. monocytogenes* serotypes (Martinez et al., 2017; Mukherjee et al., 2010; Pan et al., 2024; Rakic Martinez et al., 2020). Mukherjee and co-workers explored the ability of this insect model to discriminate between non-pathogenic and pathogenic *Listeria* species. When injected with 10^6^ CFU/larva, strains belonging to non-pathogenic species, such as *L. innocua* and *L. seeligeri* were observed to have a lower infection capacity than the *L. monocytogenes* EGD-e strain; and, although *L. ivanovii* caused a significant but slightly higher mortality than the non-pathogenic species, it presented a reduced pathogenicity efficiency compared to *L. monocytogenes* (Mukherjee et al., 2010). These results were corroborated by Martinez et al. (2017), who observed that, at the same inoculum level, the *L. monocytogenes* LS1209 reference strain displayed a LT_50_ (lethal time to kill 50% of larvae) 4 to 6 times lower than the non-pathogenic *Listeria* strains (Martinez et al., 2017).

The wax model was used to test the virulence potential of *L. monocytogenes* strains of different serotypes. The serotype 4b strain, commonly associated with clinical cases, expressed the highest larvae killing rate and was more pathogenic than the serotype 1/2a strain, usually related to food isolates. Other serotypes tested, 4a, 4c and 4d, also showed a lower pathogenic potential (Mukherjee et al., 2010). However, the study conducted by Martinez et al. (2017) showed that strains from different serotypes (1/2a, 4b, 1/2b) resulted in similar larvae mortality and identical LT_50_ at 24 h when administered at 10^6^ CFU/larva (Martinez et al., 2017). This lack of correlation between serotypes and virulence potential was in clear contrast to the findings of the former study, highlighting the importance of considering potential confounding. These may include differences in the dose of bacteria injected (10^6^ CFU/larva in the first study, whereas three different concentrations – 10^6^ CFU/larva, 10^5^ CFU/larva and 10^4^ CFU/larva – were used in the second) and the parameters analysed (Mukherjee et al. monitored the % survival along 7 days, whereas Martinez et al. focused on LT_50_ at 24 h and % mortality – not specifying its progression over the infection period). Nonetheless, it was concluded in both studies that the virulence potential of *L. monocytogenes* is dose and strain dependent, so these different results could be explained by the use of different *L. monocytogenes* strains. Another factor that could externally influence on the observed results is the larvae’s diet, since no information was available on the rearing of the larvae used in the Martinez et al. research. Previous studies have shown the importance of the diet in the larvae development, health, hemolymph volume and hemocyte concentration, which subsequently affect the immune response of *G. mellonella* (Jorjão et al., 2018; Kwadha et al., 2017). It has also been published that the diet of worms has an important impact in microbiological studies (Banville et al., 2012; Jorjão et al., 2018). Hence, standardization of diets could reduce external biases on results allowing for interlaboratory comparisons.

To date, virulence evaluation of different *L. monocytogenes* CCs using *G. mellonella* has only been performed by Cardenas-Alvarez et al. (2019). This insect model was used to compare the pathogenic potential of CC1, CC6, CC7, CC9, CC14, CC37, and CC204 strains. Briefly, differences were observed between strains from different CCs, with strains from the putatively hypervirulent CCs, CC1, and CC14, causing a reduced average survival rate (33.2 and 29.1%, respectively). Oppositely, isolates from CC9, widely accepted as hypovirulent CC, presented the highest survival rate (53.5%). In addition, the remaining CCs (6, 7, 37 and 204) showed an intermediate range of survival rates from 40 to 50%. Another parameter evaluated was the LD_50_ value (median lethal dose) – calculated from the colonies counted on plates and the number of larvae killed per day – lower values were observed for CC14, meaning that fewer cells of the pathogen are needed to kill *G. mellonella*. Cytotoxicity was also evaluated by measuring the level of lactate dehydrogenase (LDH), which is a signal of cell damage after bacterial infection. CC14 strains caused significantly less cytotoxicity than other CCs (CC6 and CC7). A positive correlation was found between LD_50_ and cytotoxicity, therefore CC14, strains by having a reduced LD_50_, also caused less injuries to host cells, which is hypothesized to be a defence mechanism to escape the host immune system and successfully spread (Cardenas-Alvarez et al., 2019). Considering these results, *G. mellonella* as an infection model, besides the capacity to differentiate non-pathogenic from the pathogenic *Listeria* species, has the potential to distinguish between virulent and attenuated *L. monocytogenes* strains from different CCs, validating its ability to discriminate the virulence potential of *L. monocytogenes*.

##### 4.1.2.2 Zebrafish model

The non-mammalian vertebrate *Danio rerio*, known as the zebrafish, is an *in vivo* model that has been gradually catching the attention of researchers for the study of infectious diseases as it meets the ideal features of vertebrate and mammalian models (Shan et al., 2015). As other models already described in this review, zebrafish is more easily applicable and economically and ethically acceptable than most mammalian models. Being a vertebrate, its morphological and genetic similarities with humans are more pronounced than with invertebrate (Pont and Blanc-Potard, 2021). In addition to the zebrafish’s large clutch dimension, *ex-utero* growth and small size, its transparency makes zebrafish a distinctive mode, allowing observation of the early stages of growth and enabling the real-time observation of bacterial infections (Pont and Blanc-Potard, 2021; Shan et al., 2015; van der Sar et al., 2004). Another interesting peculiarity is that the innate and the adaptive immune systems are temporally separated, where the primer acts singularly during early weeks while the latter is perceived just during the 4–6 weeks post-fertilization (Herbomel et al., 1999; Herbomel et al., 2001; Lam et al., 2004; Trede et al., 2004).

The use of this vertebrate model in the study of host-pathogen interactions began in 1999, when Philippe Herbomel et al., reported that primitive macrophages – which evolve during the embryo’s development and subsequently give rise to hematopoietic stem cells – develop in the zebrafish embryos at 22 h post-fertilization (Herbomel et al., 1999). Therefore, zebrafish have been used to explore host-pathogen interactions and provide new insights into the capacity of *L. monocytogenes* to cause disease in this *in vivo* model (Levraud et al., 2009; Shan et al., 2015; Zakrzewski et al., 2020). The different stages of development of zebrafish are used for research and have their advantages, but infection assays have only been performed in zebrafish’s embryos to study the association of clonal complexes with hyper-and hypovirulence of *L. monocytogenes* strains, thus, our review will merely focus on this developmental stage. Among these, Hurley et al. (2019) made use of *L. monocytogenes* strains collected from three meat and vegetable processing facilities over 4 years. Genome analysis on these isolates reported distinct virulence genotypes and grouped them into hypervirulent, hypovirulent and unknown virulence groups (Hurley et al., 2019). This classification was slightly different from that previously described by Maury et al. (2016), as the isolates commonly associated with clinical cases (strains from CC1, CC2 and CC6) were underrepresented among the isolates collected. Therefore, hypervirulent strains were selected based on the presence of additional virulence factors such as listeriolysin S from LIPI-3 or LIPI-4. Selected hypovirulent strains (CC121, CC9, CC31) harboured PMSC mutation in the *inlA* gene and some of them had a deletion on the *actA* gene, which is associated with a decrease in intracellular spread. Isolates with integral virulence factors or with minimal mutations in some genes were classified as having unknown virulence capacity (CC3). Zebrafish embryos infected with putatively hypervirulent strains presented only a 3% survival rate, followed by zebrafish embryos infected with isolates of unknown virulence (20% survival rate), while hypovirulent strains caused a higher survival rate of 53–83%, requiring 72 h post-infection to cause this decline. Using the zebrafish infection model, Hurley et al. (2019) were able to discriminate the different virulence phenotypes and confirm the previous virulence genotypes obtained by WGS (Hurley et al., 2019). Muchaamba et al. (2022) also performed infection assays using the zebrafish embryo model, comparing the virulence potential of *L. monocytogenes* strains by lineage, serotype, and clonal complex. When the strains were grouped by CC, the researchers observed virulence discrepancies by CC and strain-specific intra-clonal complex. Embryos infected with CCs that are generally considered hypervirulent showed higher mortality than isolates from CC9 or CC8. Within some CCs, such as CC1 and CC9, strain-dependent virulence variation was observed – three CC1 strains required more than 24 h post-infection to cause 100% mortality, and while two CC9 strains exhibited no virulence, the other three CC9 strains presented variable levels of virulence. The conclusion of the *in vivo* assays was that the virulence potential of this pathogen varies with genotype, serotype and strain (Muchaamba et al., 2022). Therefore, both studies confirmed the previous categorization of hypervirulent and hypovirulent *L. monocytogenes* CCs using the zebrafish embryo infection model. This underscores the model’s relevance as an *in vivo* tool for further elucidating the virulence phenotypes of *L. monocytogenes* strains.

### 4.2 *In vitro* infection models

The *in vitro* systems represent alternative processes to study bacterial virulence as they mimic the infectious mechanism, allowing, for example, screening of pathogen gene expression and how the deletion of some genes affects the behaviour of strains in physiological environments mimicking *in vivo* conditions. *In vitro* assays are based on the assumption that pathogens, such as *L. monocytogenes*, have the ability to infect hosts by attachment, invasion, multiplication and subsequent dissemination in either phagocytic or non-phagocytic cells through the production of virulence factors (Liu et al., 2007). Although these systems do not precisely replicate the full features of the host-pathogen interaction, as infectious agents may encounter unfavourable conditions and the host immune system, when compared to *in vivo* models they are less expensive, less time consuming and less ethically demanding, allowing large-scale experiments. Additionally, the ability to control experimental conditions allows to unravel favouring factors in disease. Therefore, their use is recommended for preliminary studies to find new virulence factors, after which *in vivo* models can be used on a limited scale to confirm the results (Lehr, 2002; McCoy et al., 2024; Chiang et al., 1999). For these reasons many different *in vitro* models have been developed. The standard *in vitro* system, that has been used for decades, is the 2D monolayer culture of immortalized human cells. More recently, in a bioengineering context, there has been an increase in the use of different systems based on *in vitro* and *ex vivo* models, such as organoids and 3D cell cultures, to improve the monolayer model (Taebnia et al., 2023). The choice of an appropriate *in vitro* model should focus on the definite biological issue, for example, cell lines are more adequate to study precise interaction processes of pathogens. The addition of unneeded complexity can be disadvantageous, shrouding relevant host-pathogen interactions (McCoy et al., 2024). Therefore, many tissue culture experiments to study adhesion, invasion, cell to cell spread in different cell lines, survival in macrophages, evaluation of cytotoxicity and pathogens activity upon different host environmental conditions (e.g., pH and temperature) have been reported to describe and determine novel virulence concepts of bacteria (Conte et al., 1994; Hasebe et al., 2017; Wagner et al., 2022). In *L. monocytogenes*, *in vitro* models have been used to investigate host-pathogen interactions at either an intestinal, cerebral or placental level. These models brought significant knowledge regarding the *L. monocytogenes* intracellular cycle, invasion at cell extrusion sites, the role of putative virulent genes in cell invasion, required internalins (InlA, InlB and InlP) to placental invasion, *L. monocytogenes* bacteriocins in intestinal commensals, pathogen’s routes to invade the brain and other related aspects (Banović et al., 2020; Cabanes et al., 2004; Cabanes et al., 2005; Lamond and Freitag, 2018; Pentecost et al., 2006; Rolhion et al., 2019).

#### 4.2.1 Tissue culture assays for adhesion, invasion, intracellular growth and cell-to-cell spread

In 1948 the first cell line based on subcutaneous mouse tissues was developed. Thenceforth, various mammalian cell lines have been developed and used as the primary *in vitro* model to investigate infectious diseases, since they mimic host defence mechanisms (Magdalena, 2017). In *L. monocytogenes*, the human colorectal adenocarcinoma cell line Caco-2 is one of the most popular cell models that replicate the intestinal barrier, along with HT-29, Henle-407, HeLa, and many other cell lines (Liu et al., 2007; Pizarro-Cerdá et al., 2012). Different cell lines used in listeriosis studies were represented in Table 3.

**Table 3:** Examples of cell lines used to study human listeriosis, representing the intestinal, placental and brain barriers.

Barrier	Cell line	Cell type	Reference
Intestinal	Caco-2	Human colorectal adenocarcinoma	Cajnko et al. (2015), Nightingale et al. (2005a), and Rousseaux et al. (2004)
	HT-29	Human colon adenocarcinoma	Roche et al. (2001)
	Henle-407	Human papillomavirus-related cervical adenocarcinoma	Czuczman et al. (2014)
	HeLa	Human cervix carcinoma	Bierne et al. (2005), Czuczman et al. (2014), Dhanda et al. (2021), Quereda and Pucciarelli (2014), Quereda et al. (2019), and Tham et al. (2010)
Placental	BeWo	Human placenta choriocarcinoma	Bakardjiev et al. (2004), Desai et al. (2013), Lecuit et al. (2004), Phelps et al. (2018), and Zeldovich et al. (2011)
	JEG-3	Human placenta choriocarcinoma	Bonazzi et al. (2008), Dhanda et al. (2021), and Quereda and Pucciarelli (2014)
	Primary trophoblast		Bakardjiev et al. (2004) and Lecuit et al. (2004)
Brain	HBMEC	Human brain microvascular endothelial cells	Greiffenberg et al. (2000) and Greiffenberg et al. (1998)
	HIBCPP	Human epithelial choroid plexus papilloma	Dinner et al. (2017) and Gründler et al. (2013)
	hCMEC/D3	Human cerebral microvascular endothelial cells	Ghosh et al. (2018) and Shi et al. (2024)

The main limitation of cell models is their uniformity, not truly mimicking environment and morphology of epithelial tissues where a panoply of distinct cells can be found (Hidalgo, 1996; Pearce et al., 2018). One way to overcome this limitation is to co-culture different cell lines. However, to our knowledge, this strategy has not been commonly used to study the virulence potential of *L. monocytogenes* (Laparra and Sanz, 2009; Wikman-Larhed and Artursson, 1995). Another limitation of these cell models is their cancer origin, which makes it difficult to extrapolate the data since they may not reflect the actual physiological context. Additionally, the static conditions in which these monolayers are performed lead to very rapid bacteria overgrowth, thus compromising the duration of the culture and the search for new insights about the interaction between the host and its microbiome (Rodriguez, 2018; Taebnia et al., 2023).

Summing up, these *in vitro* cell models have been widely used to study the virulence potential of *L. monocytogenes* and have contributed to expand the current knowledge of the virulence mechanism of this bacterium. Consequently, this research has led to the development of strategies to control the dissemination of listeriosis. To date, very few studies have used these models to differentiate between strains from different CCs (Domínguez et al., 2023; Møretrø et al., 2024; Schiavano et al., 2022; Wagner et al., 2022). Schiavano et al. (2022) performed adhesion and invasion assays using the Caco-2 cell line in order to do a comparative analysis between human clinical isolates and food isolates. They observed that two out of three clinical strains (from CC1 and CC101) expressed a relatively high adhesion regarding to *L. innocua*. However, the invasion efficiency was not significantly higher than that of the non-pathogenic strain. On the other hand, the food isolates showed a variable adhesion capacity, with strains from CC7, CC121 and CC1 showing significantly higher values than *L. innocua*.

For invasiveness, strains from CC7 and CC1 displayed significant higher capacity than *L. innocua*. No correlation was found between adhesion and invasion for food-derived strains. Curiously, one clinical strain (566 strain) did not show high levels of both adhesion and invasion. However, this strain belongs to CC31, which has been reported to be isolated more frequently from food than from humans. CC31 is not considered to be a hypervirulent clonal complex, as corroborated by its low invasive ability. Cases of human listeriosis caused by strains of this CC may be justified by the compromised immune system of the host (Schiavano et al., 2022). In contrast, the clinical strain tested from CC1, considered to be a common hypervirulent CC, showed an unexpectedly reduced invasion although a high level of adhesion was detected. Regarding the food isolates, the two strains with higher invasiveness belong to CC1 and CC7, which are usually associated with human cases but have also been reported in food. CC1 is highly associated with dairy and cattle products while CC7 has already been described as an intermediate CC (Lüth et al., 2020a). As expected, strains from CC9 and CC121 showed a low invasion ability. As both hypo-and hypervirulent CCs with a reduced invasion capacity were found in clinical cases, we can conclude that the state of the host immune system is very important since it may facilitate the occurrence of listeriosis. In addition, hypervirulent CCs can be found in foods, emphasizing their threat for individuals (Schiavano et al., 2022).

Wagner and his colleagues used Caco-2 cells to evaluate the invasion capacity of strains from thirteen different CCs. Strains from three CCs (CC5, CC9, and CC14) were not able to invade these cells, with only CC14 strains encoding the complete functional *inlA* gene. Strains from CC403 and CC415 were the most invasive, while strains from CC3, CC8 and CC121 were significantly less invasive. Among strains of these CCs with attenuated invasion, CC14 strains comprised all major virulent factors but still showed inefficiency at invading Caco-2 cells. Therefore, invasion and intracellular dissemination assays in Caco-2 and HEPG2 cells, both with an epithelial-like morphology, were performed with two strains of this CC. In Caco-2 cells, both CC14 strains showed a significantly reduced invasion capacity when compared to EGDe. However, only one CC14 strain showed a significantly lower invasion in HEPG2 cells. Conversely, intracellular multiplication in Caco-2 cells was significantly increased for both CC14 strains, while only one CC14 strain showed a greater intracellular spread in HEPG2 cells compared to EGDe (Wagner et al., 2022). Therefore, the virulence result of CC14 may be confusing when compared with previous findings, such as those in section 4.1.2., where it was characterized as a hypervirulent CC (Cardenas-Alvarez et al., 2019). These differences could not only be related to methodology used in the *G. mellonella* infection assays, where *L. monocytogenes* was injected directly into the hemolymph, bypassing the intestinal barrier, and thus not utilising the *inlA* and *inlB* genes, but also two distinct models (*in vitro* and *in vivo*) were used, highlighting the fact that different complexity could bring variable outcomes. Additionally, in Wagner’s study although the *inlA* and *inlB* genes were present in the genetic profile of selected CC14 strains, their expression was significantly reduced compared to *L. monocytogenes* EGDe. This reduction in expression could possibly be explained by a point mutation in the promoter region (Wagner et al., 2022). These findings of Wagner et al. (2022), point up the drawback of using solely WGS data to define virulence potentials, since presence of a virulence gene does not necessarily lead to gene expression. Besides the different approaches used, these models showed some similarities regarding the high capacity of CC14 strains to spread within the selected models.

More recently, researchers investigated the 2019 outbreak in Andalusian, Spain, caused by a strain of CC388. Adhesion and invasion *in vitro* assays were performed on A549 cell line to compare the virulence potential of this outbreak strain with strains belonging to CC1, CC4 and a reference strain from CC2 (ATCC 19115). They observed a higher adhesion of the CC388 strain compared to CC1 and the reference strains, and lower adhesion compared to CC4 strain, although no significant differences were reported. A similar pattern was observed in invasion assays, with significant differences between strains of CC1, ATCC 19115 and CC388 versus CC4. It was therefore concluded that the CC388 strain had an equal or greater virulence potential compared to other strains from hypervirulent CCs (Domínguez et al., 2023).

Concluding, 2D monolayer models have significantly advanced *L. monocytogenes* research, providing insight into key virulence properties and host-pathogen interactions. However, variations in results between different models require careful analysis. Therefore, their use must be complemented by molecular biology approaches to elucidate any unexpected phenotypic differences found by *in vitro* methods.

#### 4.2.2 Survival in macrophages

Macrophages play a crucial role in the innate immune response, using their skilled phagocytic activity to fight infection. They recognise pathogens through pattern recognition receptors (PRRs) that bind to microbial-associated molecular patterns (MAMPs) (Kumar, 2020) such as DNA, RNA, lipopolysaccharides, and lipoproteins, thereby activating the host immune response (Fitzgerald and Kagan, 2020). This interaction triggers signalling pathways that culminate in the secretion of cytokines and the process of phagocytosis. Once engulfed by macrophages, pathogens are entrapped in acidic phagosomes where antimicrobial molecules can be found (Levin et al., 2016). However, certain microbes have evolved mechanisms to evade the host’s immune defences and proliferate intracellularly, rendering macrophages ineffective in protecting against such pathogens (Galli and Saleh, 2021).

*Listeria monocytogenes* is able to survive within macrophages, as demonstrated by Tilney and Portnoy in 1989, who elucidated its mechanism of infection within these immune cells (Tilney and Portnoy, 1989). Considering this, different macrophage cell lines have been used to simulate the host barriers following bacterial intestinal invasion (Liu et al., 2007). Besides the employment of epithelial-like cells (Caco-2 and HEPG2) to evaluate *L. monocytogenes* virulence potential, Wagner et al. (2022) also used the human macrophage-like THP-1 cell line for this purpose. As CC14 strains were unable to invade Caco-2 cells, despite the presence of all key virulence genes, two CC14 isolates were used to invade and multiply intracellularly within macrophage cells. Although no significant differences in invasion capacity were observed between two CC14 strains and EGDe, intracellular multiplication was significantly increased in THP1 for both CC14 strains (Wagner et al., 2022). In 2016, Dreyer et al. conducted a study to investigate putative *L. monocytogenes* strain-associated virulence in isolates from the farm environment and diseased animals, focusing on rhombencephalitis cases where ST1 (CC1) was overrepresented. The *in vitro* assays were conducted on a bovine macrophage cell line (BoMac) and it was observed that STs associated with encephalitic infections (ST1, ST4 (CC4) and ST412 (CC412 – lineage II)) were able to invade and replicate more efficiently than those from the farm environment. Additionally, none of the isolates presented truncated InlA, which is commonly associated with virulence attenuation. Thus, although ST412 isolates from lineage II accounted for only 7% of rhombencephalitis cases (which is not a statistically significant association between ST and clinical outcome), they presented an increased virulence potential, highlighting the fact that these clinical-associated characteristics are not exclusive to lineage I isolates, raising awareness of the potential risk other CCs’. In addition, the *inlA* gene is not the only biomarker for differential virulence (Dreyer et al., 2016).

Another study conducted in 2017 aimed to test the relevance of certain virulence genes (*inlJ*1, *inlF* and *lls*) in the hypervirulence capacity of CC1 strains. The *L. monocytogenes* CC1 parental strain and its respective deletion gene mutants were compared to the EGDe (CC9) strain in different cell culture models, including macrophages. The BoMac cell line was used to mimic the intracellular phagosome environment. Despite both strains infecting all cell models, the CC1 isolate exhibited higher invasiveness than EGDe in some cell lines. For example, invasion into BoMac cells was 2.2 times higher for the strain from the hypervirulent CC. Moreover, the CC1 strain showed a significantly greater number of infection foci in BoMac cells, indicating an enhanced capacity to spread intercellularly and corroborating its stronger internalization phenotype. However, the intracellular multiplication in all cell lines was not significantly different between the two strains. *Listeria monocytogenes* exhibits a cell-specific interaction, as evidenced by the differential infection capacity observed between these two strains in specific cell types, including macrophages (Rupp et al., 2017).

In a more recent study, macrophages were used for a different comparative analysis of CCs beyond those previously mentioned, using various cell models for investigating invasion capacity, intracellular spread, and multiplication associated with potential PMSC mutations in InlA. Chalenko et al. (2022) found that InlB could modify the invasion and proliferation capacity of *L. monocytogenes* within macrophages. Consequently, they proposed to investigate whether the interaction between InlB and cell receptors would affect the intracellular infection cycle of the pathogen in these immune cells. Interestingly, this study explored the phylogenetically determined diversity of InlB to understand its impact on pathogen-cell interactions. The first step was to investigate the effective interaction between different InlB isoforms found in lineage I and II of *L. monocytogenes* strains and their two target receptors, using three distinct receptor-binding domains of InlB (idInlB) – idInlB_CC1_, idInlB_CC7_ and idInlB_CC9_ – representing different virulence potentials. The study of the interaction of idInlB-human receptors (c-Met and gC1qR) was possible by the measurement of dissociation constants using Microscale Thermoforesis technology. The results showed that the interaction between idInlB variants and human receptors differs in terms of binding strength. idInlB_cc1_ showed stronger binding to C-Met receptor compared to idInlB_cc7_ and idInlB_cc9_, while idInlB_cc9_ presented weaker binding to the gC1qR receptor, with no significance differences observed between idInlB_CC1_ and idInlB_CC7_ variants. Based on the EGDeΔinlB strain (lacking the *inlB* gene), isogenic *L. monocytogenes* strains were also constructed, namely *LmInlBCC1*, *LmInlBCC7* and *LmInlBCC9.* These strains harboured full-length internalin B isoforms that differed only in the idInlB domain. When human M1 macrophages were infected with these strains, different multiplication capacities were observed, with *LmInlBCC1* showing a significantly higher proliferation capacity compared to the other. However, no significant differences were observed regarding the cell uptake. Altogether, these results suggest that phylogenetic differences in InlB affect the ability of *L. monocytogenes* to interfere with macrophage activity. In particular, it was suggested that InlB_CC1_ efficiently overcomes immune barriers of these cells, which is consistent with the high occurrence of CC1 observed in epidemiological data (Chalenko et al., 2023).

#### 4.2.3 Organoids

Despite the unquestionable use of 2D models in studying microbe-host interactions, these models are limited in mimicking real features, such as peristaltic movements, transitions between different intestinal cells, interactions with the intestinal microbiota, and their inability to be maintained for long periods (Taebnia et al., 2023). These gaps in accurately reproducing the function and structure of the human intestinal epithelium, limit the value of 2D models. Therefore, researchers have developed more comprehensive and complex models, that do not replace monolayer models, but rather complement them by incorporating physiological components or simulating infectious disease scenarios that are difficult to assess using simpler culture methods (Taebnia et al., 2023). In the 21^st^ century, steam cells started to be cultivated to generate organoids, which have effectively bridged the gap between traditional 2D monolayer cultures and *ex vivo* models (Han et al., 2021; Sato et al., 2009; Taebnia et al., 2023).

In 2014, an important finding allowed the development of a reproducible method known as directed differentiation, which enables the specialization of leucine-rich repeat containing G protein-coupled receptor 5 (LGR5+) stem cells into various cell types, including goblet cells, enterocytes, stem cells, Paneth cells and enteroendocrine cells (Yin et al., 2014). These findings hold great promise for studying host-pathogen interactions and for investigating the responses and properties of specific cell types, such as the barrier role. Directed differentiation cannot be overstated in light of previous research showing that *L. monocytogenes* can surpass the intestinal barrier not only through enterocytes but also through M cells and goblet cells (Corr et al., 2006; Nikitas et al., 2011). Currently, organoids can be derived from cells of different species and consists of either differentiated cells, stem cells or a combination of thereof (Davies, 2018). The organoids have their apical side facing the lumen (central position), while basolateral crypt regions are directed to the outside (budding structure), mimicking the real intestinal epithelium. This reversed polarity is a challenge for microbiological research, as it is difficult to access to the lumen intestinal organoids (Huang et al., 2021). To overcome this obstacle, microinjection, mechanical dissociation techniques or “apical-out organoids” can be used (Co et al., 2019; Huang et al., 2021; Karve et al., 2017).

Many studies have used organoids to investigate host-pathogen interactions involving various microorganisms, including *L. monocytogenes* (Co et al., 2019; Huang et al., 2021; Kim et al., 2021; Roodsant et al., 2020; Zhou et al., 2022a,c). In the research of listeriosis, different types of intestinal (i.e., fetal human intestinal organoids, adult human intestinal basal-out and apical-out organoids, adult and young murine small intestinal organoids) and brain (i.e., organotypic brain slices) organoids have been used (Co et al., 2019; Guldimann et al., 2012; Hentschel et al., 2021; Huang et al., 2021; Kim et al., 2021). However, to our knowledge the use of placental organoids in the study of *L. monocytogenes* have not yet been reported (Yan et al., 2023).

Additionally, presently, the comparative analysis of different virulent *L. monocytogenes* strains using organoids has only been performed by Zhou and colleagues. However, the main objective of the authors was the study of protein changes in the host epithelium. Organoids were infected with two different strains – a “virulent strain” (serotype 1/2a) and a “low virulent strain” (serotype 4a) – and quantitative proteomic analysis of the infected mice organoids was performed (Zhou et al., 2022b). In general, it was shown that both strains were able to reduce the host’s energy metabolism, stimulate the host’s immune response and increase the expression of proteins related to adhesion and invasion, as expected. Although some differences were found between the two strains, while the virulent strain significantly activated the ferroptosis pathway – known as a cell death pathway – the attenuated strain exhibited a higher activation of the complement system, which has a crucial function in innate immune responses. Notably, both strains down-regulated nucleotide-binding oligomerization domain 2 (*NOD2*), a receptor in the NOD-like receptor signalling pathway, which is crucial for innate immune responses and can recognise pathogens through muramyl dipeptide (MDP) (Liu et al., 2023; Zhou et al., 2022b). This down regulation could potentially hamper the multiplication and protection of intestinal stem cells. So, it was expected that organoids infected with *L. monocytogenes* would show upregulation of *NOD2.* However, another study showed that germ-free mice expressed reduced levels of *NOD2*, which subsequently increased when gut commensals were added. This observation could potentially explain the reduced expression of *NOD2* in organoids as they lack gut-associated bacteria (Petnicki-Ocwieja et al., 2009). Overall, it was concluded that the immune activity and biological functions were identical in the two different strains, although some differential expression of different proteins within the pathway were observe (Zhou et al., 2022b). Considering this, the use of organoids for *L. monocytogenes* studies have been reported. However, to the best of our knowledge, no article on CC-associated virulence has been published. Thus, further research on the use of organoids as infection models for this pathogen is needed to explore their viability for comparative analysis of differentially virulent strains.

### 4.3 Molecular approaches to study virulence

The success of polymerase chain reaction (PCR) is largely due to its capacity to amplify minimal amounts of genetic material, to millions of copies in a very short time (Farrell, 2010; Mullis et al., 1986). Technical improvements have led to the quantification of gene expression through techniques such as quantitative reverse transcriptase PCR (qRT-PCR), which enables the detection and quantification of RNA products (Farrell, 2010; Zhu et al., 2020). Although qRT-PCR is considered the gold-standard for mRNA quantification, some drawbacks limit its use. Among these we highlight the additional reverse transcription (RT) step, which can introduce contamination and inhibitions, common issues in the quantification of the actual cDNA present in the sample. In addition, relative quantification requires well established reference genes that are not affected by experimental conditions, and inadequate optimization of primers and annealing temperatures can lead to non-specific sequence targeting. To address these limitations, other molecular approaches such as digital PCR (dPCR) have been developed, which allow absolute quantification without the need for reference genes and calibration curves and with a reduced probability of contamination, thus improving interlaboratory comparability (Baettig et al., 2023; Cao et al., 2020; Lei et al., 2021; Witte et al., 2016). The use of dPCR for gene expression analysis increasing, although its application is primarily limited to the detection and quantification of *L. monocytogenes* cells and biofilms (Chen et al., 2017; Grudlewska-Buda et al., 2020; Ricchi et al., 2017; Witte et al., 2016). Gene expression profiling techniques have greatly improved our understanding of how pathogens’ modulate gene transcription after interacting after interacting with the host environment, strategically recruiting their genome during the infection life cycle. Consequently, genes that exhibit differential expression during infections have captured the attention of researchers, providing insight into which virulence genes are essential for microbial pathogenicity (Shelburne and Musser, 2004).

To date, no studies using dPCR in virulence potential analysis among CCs have been reported. RT-qPCR technology is the only that has been used to study how *L. monocytogenes* strains from distinct CCs differentially express stress response genes in diverse contexts (da Silva et al., 2021; Guerreiro et al., 2022; Wambui et al., 2020; Wu et al., 2022). However, only a few research papers reporting the use of this technique to analyse differential gene expression of virulence genes associated with CCs have been published (Rupp et al., 2017; Wagner et al., 2022). Rupp et al. (2017), aimed to understand the hypervirulence of CC1, hypothesizing that it could be due to additional virulence features or genetic variation within previously studied virulence genes. They focused on three specific genes – alleles of *inlJ*1 and *inlF* and *lls* gene – two of which show some differences from lineage II strains, while the last is commonly found in lineage I CCs. Therefore, a strain from CC1 and EGDe strain (CC9) were used for intracellular (Caco-2 and BoMac cell lines) and extracellular (BHI broth) infection assays, where the gene expression of the CC1 strain was further analysed. These three virulence genes were expressed in the CC1 strain under both conditions, but the resulting PCR bands were less intense than the control genes (such as *actA* and *rrs* (16S)). However, no comparative analysis of gene expression between these two strains was explored. This study concluded that the CC1 strain was able to invade cells more effectively and exhibited an enhanced intracellular spread compared to EGDe. Nevertheless, the selected virulence genes were not found to correlate with these phenotypes, despite their strong association with the CC1 strains (Rupp et al., 2017).

Wagner and colleagues worked on the genotypic and phenotypic characterization of *L. monocytogenes* strains from the meat and salmon processing industry in Norway (Wagner et al., 2022). *In vitro* assays in Caco-2 cells and WGS analysis showed that CC14 strains lacked invasion capacity while carrying the full-length *inlA* gene. This interesting result led to a further analysis of the invasion and internalization capacity of CC14 strains using different cell lines (Caco-2, HEPG2 and THP1 cells) and the subsequent gene transcription analysis. EGDe was used as a reference. Under the conditions of Caco-2 cells infection, there were significant differences in gene expression between CC14 strains and EGDe – the expression of *inlA* and *inlB* genes was decreased in CC14 isolates, the other virulence genes (*actA*, *hly* and *prfA*) were not differentially expressed when compared to the EGDe strain. qRT-PCR was also used to study gene expression after the reconstruction of *inlA* in isolates from CC9 and CC121 that carried PMSCs mutations. Under Caco-2 cells growth conditions, the researchers observed no significant differences in the expression of *inlA*, *inlB* and *prfA* between the wildtype (WT) strains and their respective mutants. However, significant differences in the *inlA* expression were observed in both CC9 WT and one of its mutants compared to EGDe. The CC121 *inlA* reconstructed mutants showed no significant differences in gene expression when compared to EGDe, indicating successful gene reconstruction (Wagner et al., 2022). This study highlights the need for analysis of gene expression, since strains from CC14, although harbouring the full-length *inlA* gene, showed reduced expression of this virulence gene. We can conclude that the qRT-PCR technique is an essential tool to obtain further information about the virulence potential of *L. monocytogenes* strains.

## 5 Discussion

Characterization of the virulence potential within *L. monocytogenes* strains is essential for effective risk assessment and to reduce the human and economic losses associated with listeriosis. Multi-locus sequence typing, combined with epidemiological data, has facilitated the identification of more or less virulent CCs, helping researchers to refine their understanding of the infection patterns of this pathogen. However, despite advances in CC characterization, the specific virulence markers that confer distinct disease-causing capacities remain poorly understood.

This review highlights the limited use of different infection models to study the virulence potential of *L. monocytogenes* CC. While some models, such as *G. mellonella* larvae, have not been extensively studied, results suggest variability in results depending on the model used. Some studies successfully distinguish hyper-and hypovirulent CCs, while others show inconsistent results, suggesting a strain-dependent characterization of infection risk within CCs. The strain-dependent nature of *L. monocytogenes* virulence potential, together with highly variable host susceptibility and evidence that virulence may be influenced by the food matrix, limits the usefulness of subtypes. Grouping them into Clonal Complexes (CCs) may mask relevant differences in their pathogenicity. Additionally, we highlight the fact that a standard infection model for studying the virulence potential of *L. monocytogenes* CCs has not yet been established. This lack of standardization difficult comparisons between dies, as variations in experimental conditions, infection doses and techniques may lead to different virulence outcomes. Despite these challenges, the use of different approaches to validate previous findings is recommended, and improvements to existing models, mainly 3D systems, are ongoing.

The presence or absence of virulence genes alone is not sufficient to determine pathogenicity, highlighting the multifactorial nature of listeriosis. Consequently, the achievement of a zero risk infection remains elusive and requires a comprehensive understanding of the host immunity and pathogen virulence machinery. Despite ongoing efforts, a reliable virulence biomarker capable of differentiating attenuated strains has yet to be identified, underscoring the importance of continued research and refinement of infection models to advance our understanding and control of listeriosis. To conclude, we would like to highlight a recent study that used artificial intelligence to predict the virulence potential of *L. monocytogenes* at the subspecies level using WGS datasets. The use of the pan-genome showed the best predictive results, pointing up the possible value of pan-genome related genes in virulence. Although the authors acknowledge some of the limitations of their models, such as the difficulty of generalizing data based on WGS from only three countries, which rises concerns about the application of these predictive machine learning models in other regions (Gmeiner et al., 2024), these findings show that innovative technologies are being explored for future risk assessment. Additionally, besides the limitations of the current use of subtype classification for characterizing *L. monocytogenes* virulence, their use cannot be ruled out as more is learned about risk variability. In the future, standardized risk assessment models may serve as valuable tools for the food industry in risk management upon *L. monocytogenes* contamination.

## Footnotes

^1^http://bigsdb.pasteur.fr/
